# Exploring the structural changes in nitrogen-fixing microorganisms of rhizosheath during the growth of *Stipagrostis pennata* in the desert

**DOI:** 10.1042/BSR20201679

**Published:** 2021-04-14

**Authors:** Yongzhi Tian, Xiaolin Ma, Yuanting Li, Cong Cheng, Dengdi An, Fengwei Ge

**Affiliations:** 1 Xinjiang Key Laboratory of Special Species Conservation and Regulatory Biology, College of Life Science, Xinjiang Normal University, Urumqi 830054, Xinjiang, China; 2 College of Life Sciences, Nanjing Agricultural University, Nanjing 210095, Jiangsu Province, China

**Keywords:** nifH Sequencing, Ntrogen-fixing microorganisms, Rhizosheath, Stipagrostis Pennata

## Abstract

**Purpose:** Rhizosheath is an adaptive feature for the survival of *Stipagrostis pennata* in desert systems. Although microorganisms play important ecological roles in promoting the nitrogen cycle of rhizosheath, the diversity and function of nitrogen-fixing microorganism communities have not been fully understood.

**Materials and methods:** Therefore, the aim of the present study is to explore the nitrogen fixation ability of rhizosheaths and the changes in abundance of nitrogen-fixing microorganisms at different growth periods of *S. pennata*. We sequenced the *nifH* gene through sequencing to identify the structure and diversity of nitrogen-fixing microorganisms of *S. pennata* at different growth periods of rhizosheaths.

**Results:** A total of 1256 operational taxonomic units (OTUs) were identified by nifH sequence and distributed in different growth periods. There were five OTUs distributed in each sample at the same time, and the abundance and diversity of microorganisms in fruit period were much higher than those in other periods. Mainly four phyla were involved, among which *Proteobacteria* was the most abundant in all groups.

**Conclusions:** In general, the present study investigated the abundance and characteristics of nitrogen-fixing microorganisms of rhizosheaths in different growth periods of *S. pennata*. It also may elucidate and indicate that the structure of nitrogen-fixing microorganisms of rhizosheaths in different growth periods of *S. pennata* had changed.

## Introduction

Desert is one of the harshest terrestrial ecosystems on earth, characterized by high solar radiation level, low rainfall and extremely high temperature. In addition, the character of desert soil is low water retention, low nutrition level and high salinity [1]. Desert ecosystems cover most of the earth’s land surface and are characterized by extremely low productivity and limited availability of water and nitrogen (N) as always [2]. Due to human activities and climate change, a larger proportion of these arid lands are facing the threat of continuous desertification [3]. Desert ecosystems are generally considered to be inanimate habitats under extreme environmental conditions, despite the fact that they are colonized by a few microorganisms [4]. Desert ecosystems may change due to global climate change and nitrogen (N) deposition. The effects of precipitation and increased nitrogen deposition on plant growth and nitrogen cycle largely depend on nitrogen distribution and nitrogen recovery efficiency in plant–soil ecosystems, but the researches on desert ecosystems are limited [5].

In desert ecosystems, the distribution of higher plants and animals is limited by extreme environmental conditions in deserts or arid areas. Microorganisms are considered to be the main driving force of ecosystem services and can regulate key ecosystem processes [6,7]. Gramineae plants growing in sandy/rocky desert soil have developed a root system called ‘rhizosheath’ as a trait to adapt to drought and absorb nutrients [8]. The rhizosheath is defined as a part of the soil that physically adheres to the root system and can encase the entire root system of certain plants [9]. Rhizosheath-related microorganisms of desert plants can promote plant growth and enhance stress tolerance, especially nitrogen-fixing related microorganisms [10,11]. *S. pennata* (Gramineae) has advantages in effectively resisting long-distance diffusion and occupying the surrounding optimized environment [12]. Our previous experimental results show that the strategy of *S. pennata* against harsh environment is related to the nitrogen fixation ability of root sheath [13]. Nitrogenase is a protein complex gene encoded by nifH, nifD and nifK [14]. NifH is the most widely used part to study the diversity and composition of nitrogen-fixing microbial communities [15]. Some factors may also affect the composition of bacterial communities that actually express nifH and/or the number of nifH transcripts [16].

The sequence of nifH is highly conserved, so nifH could be used to study the diversity of nitrogen-fixing microorganisms in soil. In the present paper, we identified the sequence of nifH in rhizosheath at different growth periods of *S. pennata* by nifH sequencing. Based on the species and quantity of nitrogen-fixing microorganisms obtained from rhizosheaths, the possible factors for *S. pennata* to become the pioneer population in desert were evaluated.

## Materials and methods

### Collection of *S. pennata* rhizosheaths

The samples of *S. pennata* rhizosheaths were collected from same hinterland of Junggar Basin in Xinjiang and the representativeness of samples in different growth periods was ensured. The randomly collected samples were as follows: Returning Green Period (A) Sample Numbers: TYZ-10, TYZ-48, TYZ-51; Flowering Period (B) Sample Numbers: TYZ-8, TYZ-24, TYZ-27; Filling Period (C) Sample Numbers: TYZ-3, TYZ-43, TYZ-45; Fruit Period (D) Sample Numbers: TYZ-14, TYZ-21, TYZ-23; Withering Period (E) Sample Numbers: TYZ-6, TYZ-36, TYZ-37, i.e. three duplicate samples were set for each growth period.

### The extraction of DNA and amplification of nifH

Power Soil™ DNA Isolation kit (MOBIO, U.S.A.) was used for total DNA extraction according to the manufacturer’s instructions. The concentration and quality of DNA were detected by NanoDrop spectrophotometer. The nifH was amplified by using polF/polR primers through PCR. PCR products (Liu, Liberton et al. 2018) were purified by using Ambion DNA-free kit (Life Technologies, U.S.A.). All molecular experiments were done in the Key Laboratory of Special Environment Biodiversity Application and Regulation in Xinjiang, Key Laboratory of Plant Stress Biology in Arid Land, and The Key Discipline Biology of Xinjiang Normal University.

### NifH sequencing

The PCR product was purified and cloned into pGEMs-T Easy Vector System kit (Promega, France), and the positive clone was sequenced (Biofidal, Vaulx-en-Velin, France). According to the requirements of Illumina library preparation scheme, sequencing samples were prepared by TruSeq DNA kit, then applied to Illumina Miseq system, sequenced by Reagent Kit v2 2×250 bp.

### Statistical analysis

Sequencing data were processed using qiime pipeline-version 1.7.0 (http://qiime.org/). The sequence with high quality was used for subsequent analysis. Uchime algorithm was used to check the aligned nifH gene sequence, and the sequences were removed from the dataset before the operational taxonomic unit (OTU) table was constructed. High-quality sequences were clustered into OTUs with 97% identity or threshold. Diversity analysis of α and β diversity was calculated, and metastats group significant difference analysis was used to access whether OTU had differences by Kruskal–Wallis rank sum test. Species with significant differences were analysed by Linear Discriminant Analysis (LDA) Effect Size in abundance between groups.

## Results

### Identification of structure and diversity of nitrogen-fixing microorganisms in rhizosheaths by nifH sequencing

**Figure 1:**
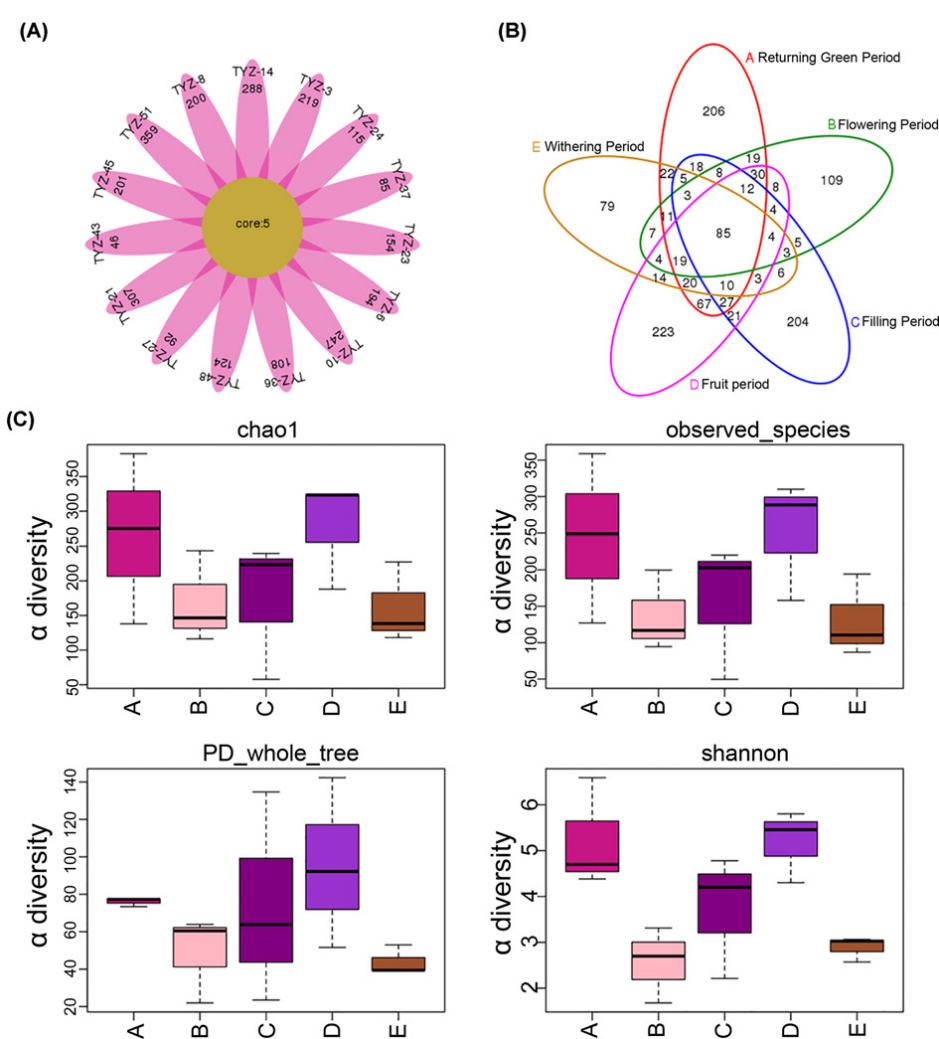
The structure and diversity of OTU (**A**) Petal map of OTU distribution. Each petal represents a sample, the core number in the middle represents the number of OTUs common to all samples, and the number on the petal represents the number of OTUs unique to the sample. (**B**) Venn diagram of OTU distribution. Different colors represent different groups, overlapping areas of circles of different colors are the same OTU in several groups, and non-overlapping parts are unique OTUs. (**C**) α diversity index box chart. The box chart mainly contains five data nodes. A group of data from large to small was arranged, and its upper edge, upper quartile, median, lower quartile and lower edge were calculated, respectively. The abscissa is the grouping name and the ordinate is the α index. Group A: Returning Green Period, Group B: Flowering Period, Group C: Filling Period, Group D: Fruit Period, Group E: Withering Period.

*S. pennata*, as a pioneer species of desert plants, the nitrogen-fixing microbial environment of rhizosheath plays important role. Therefore, we sequenced the nifH through sequencing the nifH gene and identified the structure of the species. A total of 493225 valid readings were obtained from 15 samples (Supplementary Table S1). Statistical analysis of the distribution of high-quality sequences showed that most sequences were aggregated at 320–360 bp (Supplementary Figure S1). Further, 1256 OTUs of all metagenomics samples were obtained after using 97% similarity truncation, which mean that the species richness of nitrogen-fixing microorganisms identified by nifH was very high (Supplementary Table S2). Statistical analysis showed that not only each sample had its own unique OTUs, but also five OTUs were distributed in each sample simultaneously (Figure 1A). The distribution of OTUs among the sample groups in the five periods of *S. pennata* was also shown by veen diagram (Figure 1B). OTUs were most abundant in fruit period (Group D), followed by green period (Group A) and filling period (Group C). Furthermore, α diversity measurements were utilized for root sheath microorganisms at each period of *S. pennata* (Figure 1C). Comprehensive analysis clarified that microbial abundance in fruit period was much higher than that in other periods. The sparse curves of all samples were on the brink of the platform, indicating that the diversity of microbial communities was well captured (Supplementary Figure S2).

### Taxa of nitrogen-fixing microorganisms in rhizosheaths at different periods

**Figure 2:**
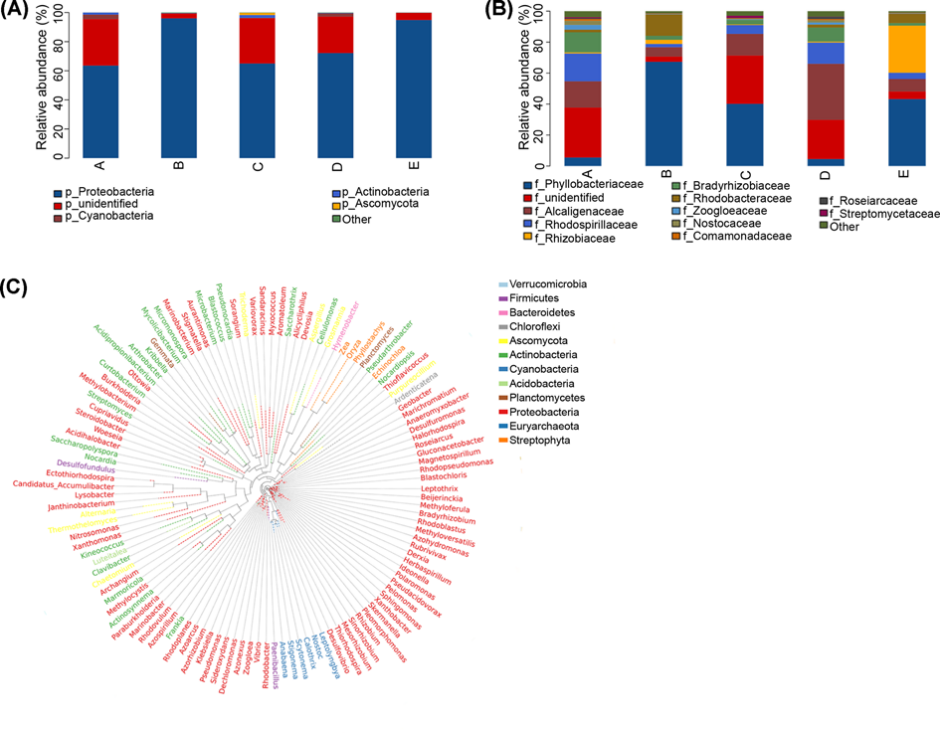
Species annotation and genetic identification (**A**) Histogram of species composition analysis. The abscissa is the grouping name, and the ordinate is the relative abundance of species in the sample. The figure shows species information of phylum with relative abundance over 1%. (**B**) Histogram of species composition analysis. The abscissa is the grouping name, and the ordinate is the relative abundance of species in the sample. The figure shows species information of families with relative abundance of more than 1%. (**C**) Genus level evolutionary tree. The representative sequence of OTU with the largest abundance is selected as a unit of genus to build trees, and the outer circle of the evolutionary tree shows the relative abundance of each genus in different groups. The length of the color block represents the relative abundance.

Approach to know the types of nitrogen-fixing microorganisms, we annotated the OTUs obtained by nifH sequencing. The microbial community was mainly composed of four phyla, encompassing *Proteobacteria*, *Cyanobacteria*, *Actinobacteria* and *Ascomycota*, of which *Proteobacteria* was the most abundant in all groups (Figure 2A). There were also a small number of microorganisms that have not been annotated. *Ascomycota* had a higher proportion in the filling period than other groups, and *Cyanobacteria* had a higher abundance in the turning green period and fruit period. The family of 11 nitrogen-fixing microorganisms was associated with rhizosheaths (Figure 2B). *Physiologicae* was the most abundant in flowering period (Group B), *Alcaligenaceae* was in fruit period, while *Rhizobiaceae* was in withering period (Group E).

The relationship between nitrogen-fixing microbial communities was more formally evaluated by distinguishing phylogenetic trees of major nifH types (Figure 2C). Compared with other periods, nitrogen-fixing microbial community indicated higher phylogenetic diversity in fruit period. *Mesorhizobium* genus existed in most samples of five periods, meanwhile *Azohydromonas* and *Azospirillum* also existed in rhizosheaths of five periods. These three species belong to *Proteobacteria* phylum.

### Overall changes in community structure of nitrogen-fixing microorganisms in rhizosheaths

**Figure 3:**
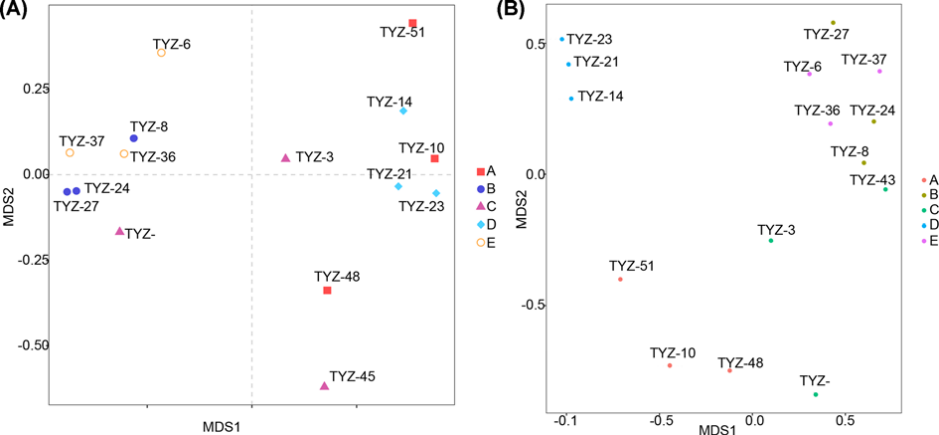
β diversity analysis of OTU (**A**) Principal component analysis (PCA) based on OTU level. Points of different colors or shapes represent different sample grouping situations. (**B**) NMDS analysis based on OTU level. Different groups in the figure are represented by dots of different colors. The closer the dots are, the more similar the samples are.

To better distinguish nitrogen-fixing microbial communities in different periods in rhizosheaths of *S. pennata*, we entered to conduct β diversity analysis on nitrogen-fixing microbial communities identified by nifH. Bacterial communities differed between samples at different times (Figure 3A). Although OTU changed highly in different samples, samples from each period usually formed clusters, especially in flowering and fruit periods. According to the results of NMDS, the difference of root sheath OTUs were significantly different between different periods (Figure 3B). The difference of OTU group in fruit period was not obvious, but it was much larger than other groups. It was further confirmed that there were obvious structural changes in nitrogen-fixing microorganisms in fruit period, and there was a low similarity between them and other periods.

### The specifity and relativity of nitrogen-fixing microorganisms in rhizosheaths at different periods

**Figure 4:**
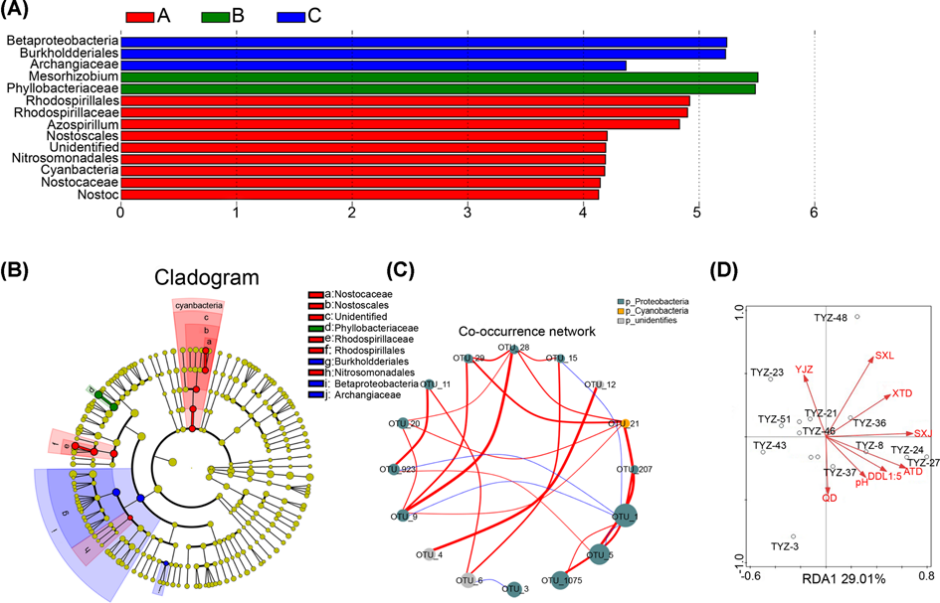
Specificity and correlation of nitrogen fixation-related microorganisms in different growth periods (**A**) Histogram of LDA distribution based on LEfSe analysis of classification information shows species with LDA score greater than 3. The length of the histogram represents the magnitude of the significant difference in species. (**B**) Evolutionary bifurcation diagram of LEfSe analysis based on classification information. Circles radiating from inside to outside represent the classification level from phylum to genus. The diameter of the circle is proportional to the relative abundance. Species with no significant difference were uniformly colored yellow, and the different species Biomarker followed the group for coloring. (**C**) The gate level network interaction map of OTU in the first 20 abundance. The size of dots represents the abundance, and the line thickness represents the correlation. The color of the dots represents the phylum to which it belongs, the red line indicating a positive correlation, and the blue line indicating a negative correlation. (**D**) Double mapping of canonical correlation analysis (RDA) environmental parameters and *nifH* gene.

LDA was utilized to reduce the dimension of data and evaluate the influence of species with significant differences (Figure 4A). OTUs with significant differences were obtained in the green returning period, flowering period and fruit period. *Rhodospirillales*, *Mesorhizobium* and *Betaproteobacteria* have great influence on nitrogen fixation in the green returning period, flowering period and fruit period, respectively. Secondly, classification information-based LEfSe analyzed data from nifH sequencing to detect the community composition of nitrogen-fixing microorganisms (Figure 4B). The results elucidated that the abundance of representative nitrogen-fixing microbial communities in the green returning period and fruit period was extremely higher than that in the flowering period. Finally, the OTUs of the top 20 absolute abundance of all samples were selected for correlation analysis with phylum level annotation results through Spearman’s test method (Figure 4C). Two kinds of microorganisms were identified in these OTUs, including *Cyanobacteria* and *Proteobacteria*; *Proteobacteria* had the highest microbial abundance. OTU1 had the most correlation with other OTUs, encompassing positive and negative correlation. The change of microbial community associated with nitrogen fixation in different periods is also closely related to environmental change. Canonical correlation analysis (RAD) shows that the environmental factors are correlated with the total microorganism (Figure 4D). Specifically, flowering was correlated most strongly with ammonium nitrogen (ATD), fruit was strongly influenced by YJZ (soil organic matter). The results indicated that these microorganisms exert biological functions such as nitrogen fixation through mutual influence, which regulate the abundance of nitrogen-fixing microorganisms in *S. pennata* during different periods of rhizosheaths.

## Discussion

As we know, desert soil is characterized by harsh environmental conditions, such as extreme temperature, dryness, high soil salinity, low nutrition level, high ultraviolet radiation level and physical instability [17]. Plants are identified as an important driving factor of the diversity of functional gene pool in arid soil ecosystems. Under desert conditions, pioneer desert plants usually interact closely with soil microorganisms in a high degree to achieve appropriate survival strategies [18]. Rhizome deposition occurs around and along the entire root length in the stressed environment of dry soil and drought (e.g. desert and gravel plain soil), forming compact rhizosphere structures associated with plant stress resistance in some species [19]. Nitrogen-fixing microorganisms in desert play an indispensable role in the development and growth of plants. Microbial nitrogen fixation is catalyzed by nitrogenase, a complex metalloenzyme found only in prokaryotes [20]. Hence, we identified nitrogen-fixing microorganisms with nitrogenase activity by sequencing the *nifH* gene of *S. pennata*’s rhizosheath in the desert. A total of 1256 OTUs were identified in different periods of *S. pennata*, indicating that there were large number of nitrogen-fixing related microorganisms involved in plant growth in rhizosheaths. This may be a potential factor for *S. pennata* to become a pioneer plant in the desert. However, the identified OTU was also distinctive in different growth periods, regardless of the abundance or diversity of microorganisms.

Approach to understand the relationship between changes in the structure of nitrogen-fixing microbial communities and plant growth, we compared and analyzed nitrogen-fixing microbial communities in different growth periods. *Cyanobacteria* was abundant in the green turning period and fruit period, which was widely distributed in marine, aquatic and terrestrial ecosystems, and played an important role in the global nitrogen cycle [21]. There were also other studies that access the nitrogenase activity of *Cyanobacteria* by sequencing the *nifH* gene [22]. The CNFR1/CNFR2 protein family was the main activator of *Cyanobacteria* nitrogenase gene expression [23]. In addition, *Alcaligenaceae*, which was highly abundant in the fruit period, had an essential influence on the nitrogen fixation of rhizosheaths. Some studies had confirmed the nitrogen fixation nutritional properties of *Alcaligenaceae* by Western blot analysis of nicotinase reductase and nifH amplification [24]. *Rhizobiaceae*, which is the principal nitrogen-fixing microorganism in the root nodule structure of leguminous plants, had the highest abundance in withering period [25]. The above results showed that the number and types of nitrogen-fixing microorganisms changed in different growth periods. It is known that plants recruit microbial communities by releasing root exudates into rhizosphere, thus providing the main source of carbon and nutrients. The composition of root exudates depends on soil type, plant genotype, growth period etc [26]. The microbial community recruited formed the root microorganism [27,28]. So that, we believed the growth process of *S. pennata* and the microorganisms in the rhizosphere interacted with each other to achieve better results of adapting to the environment and good growth state.

Rhizosphere association between nitrogen-fixing microorganisms and plants has been the main driving force that allowed organisms to diffuse in biosphere, occupy new niche and adapt to various environmental pressures. Not surprisingly, *Mesorhizobium*, *Azohydromonas* and *Azospirillum* dominated the living microenvironment of *S. pennata*. These ubiquitous phyla were found in the desert sands around the world [29]. Through diversity analysis, it was clarified that the structure of nitrogen-fixing related microorganisms in fruit period was significantly better than that in other periods. As a representative community of nitrogen-fixing related microorganisms in fruit period, *Betaproteobacteria* could provide nitrogen for the growth of most plants, including leguminous and non-leguminous plants [30,31]. Some genus in *Betaproteobacteria* could grow together with corn, rice and wheat to contribute to improving plant growth, indicating that rhizosheath had greater ability to aggregate nitrogen-fixing microorganisms in fruit period, which might be related to its secretion. In addition, we found that the flowering and fruit periods were affected by ammonium nitrogen (ATD) and soil organic matter (YJZ), respectively. The distribution of microorganisms has always been closely related to the living environment [32,33].

In the present study, large numbers of rhizosheath microorganisms related to nitrogen fixation were identified by sequencing the *nifH* gene. Further identifying that the structure and quantity of microorganisms related to nitrogen fixation in rhizosheaths during fruit period was more advantageous. The in-depth profile of microorganisms related to nitrogen fixation at different growth periods of *S. pennata* helped us to understand the interaction between plants and microorganisms in the desert environment.

## Conclusion

In the present study, large numbers of rhizosheath microorganisms related to nitrogen fixation were identified by sequencing the *nifH* gene. Further identifying that the structure and quantity of microorganisms related to nitrogen fixation in rhizosheaths during fruit period was more advantageous. The in-depth profile of microorganisms related to nitrogen fixation at different growth periods of *S. pennata* helped us to understand the interaction between plants and microorganisms in the desert environment.

## Supplementary Material

Supplementary Figures S1-S2 and Tables S1-S2

## Data Availability

The original contributions presented in the study are included in the supplementary material.

## Abbreviations

LDA: linear discriminant analysis
LEfSe: inear discriminant analysis Effect Size
NMDS: Non-metric multidimensional scaling
OTU: operational taxonomic unit
