# Supplementary material for: Exploring the structural changes in nitrogen-fixing microorganisms of rhizosheath during the growth of Stipagrostis pennata in the desert
Source: Biosci Rep. 2021 Apr 14;41(4):BSR20201679. doi: 10.1042/BSR20201679 (PMC8047386; doi:10.1042/BSR20201679)
Supplement: Supplementary Figures S1-S2 and Tables S1-S2 [file BSR-2020-1679_supp.pdf]

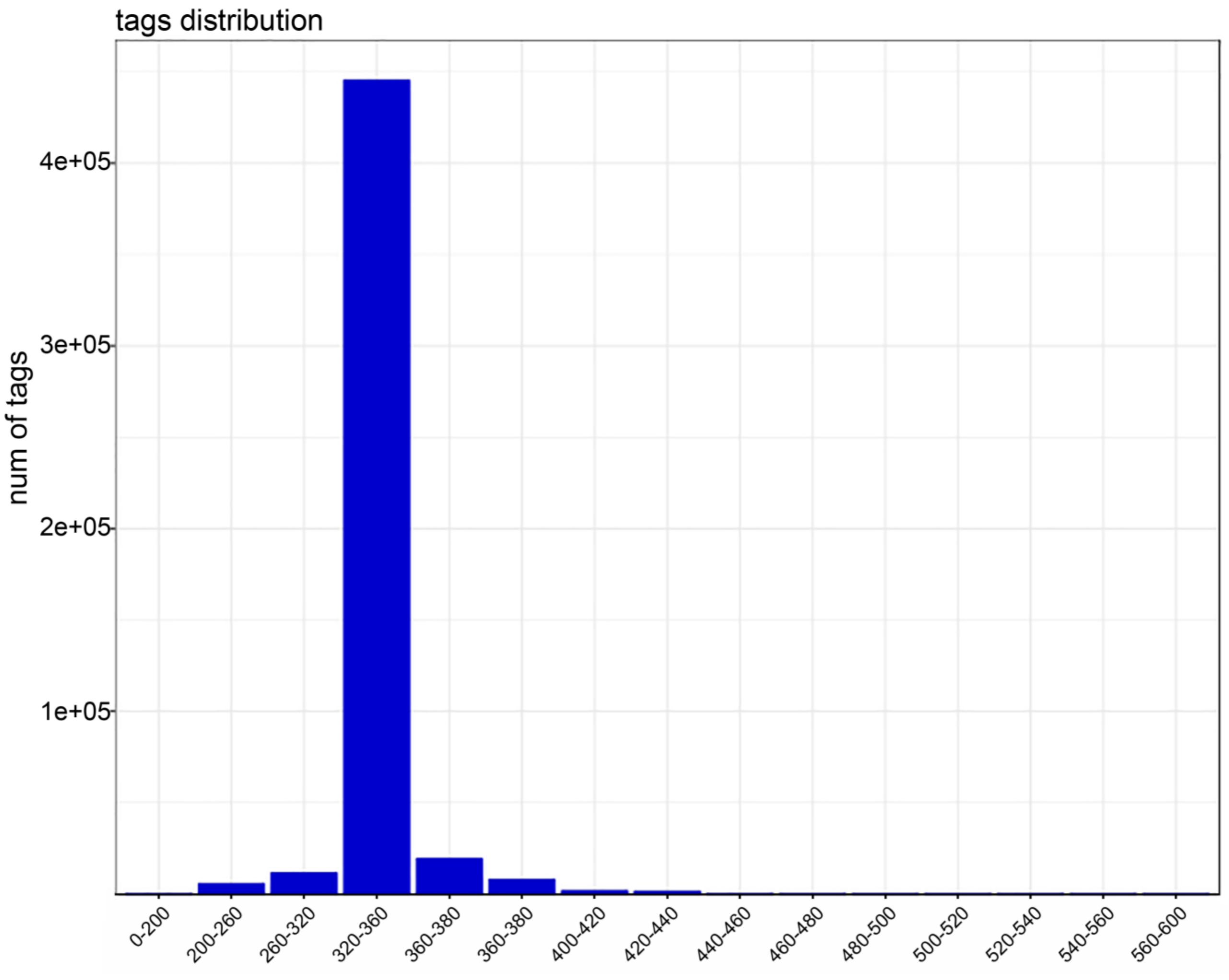
tags distribution
num of tags
4e+05
3e+05
2e+05
1e+05
0-200
200-260
260-320
320-360
360-380
360-380
400-420
420-440
440-460
460-480
480-500
500-520
520-540
540-560
560-600

Figure S1. Length distribution of high quality sequences. The abscissa is the sequence length gradient and the ordinate is the tags number.

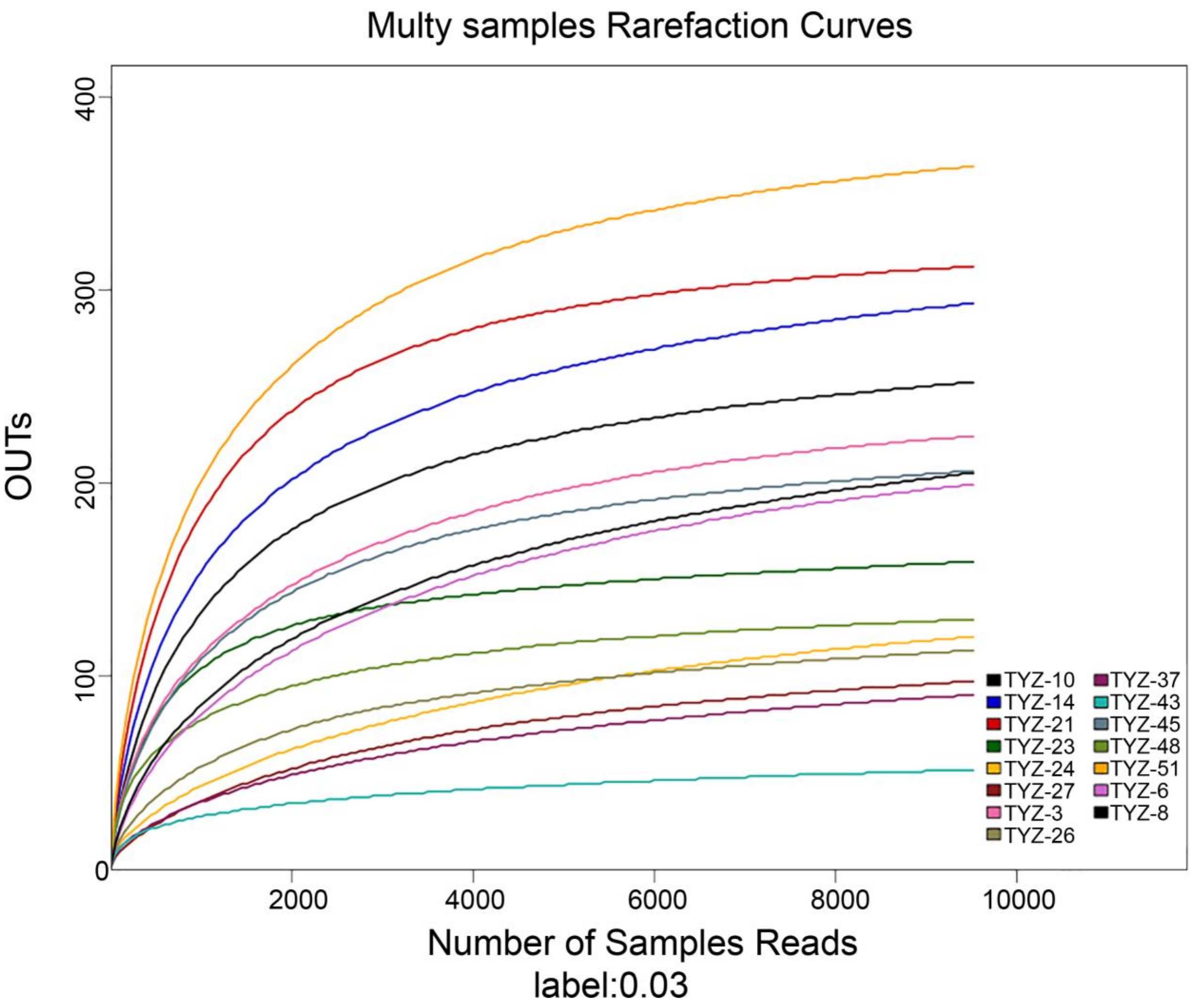

Multy samples Rarefaction Curves
OUTs
0
100
200
300
400
2000
4000
6000
8000
10000
Number of Samples Reads
label:0.03
TYZ-10
TYZ-14
TYZ-21
TYZ-23
TYZ-24
TYZ-27
TYZ-3
TYZ-26
TYZ-37
TYZ-43
TYZ-45
TYZ-48
TYZ-51
TYZ-6
TYZ-8

Figure S2. Sample dilution graph. The abscissa is the randomly selected sequencing data quantity, and the ordinate is the observed OTU quantity. Lines of different colors represent different samples.

Table S1. Statistics of Stitching Results

SampleID	raw_tags	clean_tags
TYZ-10	17987	16986
TYZ-14	18460	17627
TYZ-21	13584	11841
TYZ-23	23152	21399
TYZ-24	54795	53509
TYZ-27	74132	73282
TYZ-36	53648	50630
TYZ-37	55121	53153
TYZ-3	18336	17507
TYZ-43	61600	61540
TYZ-45	31430	31229
TYZ-48	25828	25559
TYZ-51	13626	12413
TYZ-6	27279	26228
TYZ-8	21140	20322

Table S2. OTU Number Statistics for Single Sample

SampleID	Final_tags	OTUs
TYZ-10	9518	252
TYZ-48	9518	129
TYZ-51	9518	364
TYZ-8	9518	205
TYZ-24	9518	120
TYZ-27	9518	97
TYZ-3	9518	224
TYZ-43	9518	51
TYZ-45	9518	206
TYZ-14	9518	293
TYZ-21	9518	312
TYZ-23	9518	159
TYZ-6	9518	199
TYZ-36	9518	113
TYZ-37	9518	90
